# Safety and effectiveness of single ProStyle™/ProGlide™ for aortic endovascular procedures: single-style study

**DOI:** 10.3389/fcvm.2025.1559131

**Published:** 2025-07-03

**Authors:** Rocco Cangiano, Alessia Di Girolamo, Marta Ascione, Francesca Miceli, Carola D’Amico, Andrea Molinari, Ada Dajci, Antonio Sterpetti, Giovanni Gagliardo, Luca di Marzo, Wassim Mansour

**Affiliations:** ^1^ Vascular and Endovascular Surgery Division, Department of General Surgery and Surgical Specialties, Policlinico Umberto I, “Sapienza” University of Rome, Rome, Italy; ^2^ Department of General Surgery, Policlinico Umberto I, “Sapienza” University of Rome, Rome, Italy

**Keywords:** vascular access, vascular access closure devices, aortic endovascular procedure, pre-close technique, vascular-diagnosis

## Abstract

**Background:**

The Perclose ProStyle™/ProGlide™ Suture-Mediated Closure and Repair (SMCR) System is designed to close the common femoral artery (CFA) access during percutaneous endovascular procedures. Instructions for use (IFU) recommend the use of at least two devices, per single access, and the pre-close technique for arterial sheath sizes greater than 8 F. Besides, recent clinical studies suggest that a single ProStyle™/ProGlide™ pre-implantation can safely close percutaneous access for larger diameters.

**Aim:**

The purpose of this study was to analyse the efficacy of a single pre-implanted ProStyle™/ProGlide™ in closing the Common Femoral Artery (CFA) access site in patients undergoing aortic endovascular procedures using sheaths with diameter 12–16 French (F).

**Methods:**

We performed a prospective study including 72 consecutive patients who underwent aortic endovascular surgery from December 2022 to June 2024 in our University Hospital. In this group, only a single pre-implanted ProStyle™/ProGlide™ was used to close the access site in the CFA after using sheaths with diameters 12–16 F. The primary endpoint was technical success, defined as the absence of intraoperative open surgical conversion. The secondary endpoint was clinical success, defined as the absence of bleeding, pseudoaneurysms, and arteriovenous fistulas in the peri and postoperative period. We compared the results of this group with a cohort of patients in whom two ProStyle™/ProGlide™ were used to close the access site in the CFA.

**Result:**

Technical success was achieved in all cases (100%). Clinical success was achieved in 98% of cases. Only two minor bleedings occurred: one resulted in a small pseudoaneurysm, completely thrombosed 48 h after the procedure. One patient suffered from CFA dissection, requiring an open surgical endarterectomy. There were no statistically significant differences of clinical and technical success rates between the two groups.

**Conclusions:**

This study demonstrates that a Single ProStyle™/ProGlide™ preimplantation can be safe and effective in the closure of vascular accesses up to 16 Fr, with a low complication rate.

## 1 Introduction

Endovascular procedures have become the preferred treatment for patients with complex aortic diseases (1–4). Percutaneous access for EVAR (PEVAR) (5) is an accepted alternative to traditional EVAR with surgical cut-down (SEVAR), reducing procedure's invasiveness (5–8) and surgical site complications (6). Technical success rate of PEVAR has improved over the years, from 62%–100% (9–16). Most studies have utilised the Prostar™ (Abbott Perclose, Redwood City, Calif) closure device; the newest version of this device, the ProStyle™/ProGlide™ device, presents higher possibilities for successful closure (12, 17, 18).

Improvements in technical details (18–20) such as percutaneous devices capable of closing ever larger accesses and endovascular devices have led to wider use of total percutaneous techniques of endovascular procedures (21), even in challenging anatomies (20), with positive midterm outcomes (7). Several techniques are available for vascular haemostasis, including suture-based, collagen-based, clip-based devices or their combination (19, 22). Femoral calcification seems to be the only predictor of percutaneous access failure (23).

Percutaneous approach is usually performed under local anaesthesia, contributing to reduced operative time, hospital stay, and lower perioperative complications related to general anaesthesia and surgical cutdown (24–30).

The aim of this study was to analyse the efficacy of using a single ProStyle™/ProGlide™ in closing access sites with sheath diameters greater than 12 French (F) (4. 7 mm) up to 16 F (5. 3 mm), in patients undergoing endovascular aortic procedures.

## 2 Materials and methods

Two groups of patients were compared: patients requiring a single ProStyle™/ProGlide™ per percutaneous femoral artery site (OneStyle group), and patients who underwent standard protocol ProStyle™/ProGlide™ with two preimplanted devices per femoral access site (Control Group). Data were collected prospectively for the first group, and they were compared with data of the second group, which were collected retrospectively.

The first group included patients who underwent elective percutaneous endovascular aortic procedures from December 2022 to June 2024 at a tertiary University hospital, while the control group consisted of patients who underwent the same endovascular procedures from 2018–2023. Patients were matched 1–1 according to age, sex, clinical conditions, and type of endovascular surgery.

Exclusion criteria included femoral diameter of less than 5 mm; different access vessels than the CFA; access size sheath <12 F and >16 F; the Modified Peripheral Arterial Calcium Scoring System (mPACSS) (31) score of 2; arterial stenosis of more than 50%, previous vascular grafts reconstruction with bypass surgery or stenting of the femoral artery (Table 1).

**Table 1:** Exclusion and inclusion criteria of the study.

Access characteristics	Exclusion Criteria	Inclusion Criteria
Diameter CFA	<5 mm	>5 mm
Sheat size	<12 F, >16 F	12–16 F
mPACSS	+2 point	0 point
	Calcification in ≥50% or annular calcification in ≥25%	No calcification or <25%
		+1 point
		Calcification between 25 and 50%
Thrombosys/plaque	Severe stenosis	No or mild stenosis
	>50%	<50%
Previous vascular graft reconstruction of CFA	Artery stenting, prosthetic or venous bypass	None (native vessel)

We analysed technical and clinical success using a single ProStyle™/ProGlide™ to close the access site in the Common Femoral Artery (CFA) with sheaths diameter from 12 Fr up to 16 F.

All patients were followed postoperatively with a Duplex Ultrasound (DUS) examination and computed tomography angiography (CTA) performed at 1 month.

The primary endpoint of this study was technical success, defined as the correct device implantation and the absence of intraoperative open surgical conversion. Secondary endpoint was the clinical success, defined as the absence of bleeding, pseudoaneurysms, and arteriovenous fistulas in the postoperative period.

Outcomes of interest were classified according to Bleeding Academic Research Consortium (BARC) criteria (32) that were subdivided into life-threatening or disabling bleeding (bleeding in a critical organ, hypovolemic shock, or hypotension requiring vasopressors), major bleeding (overt bleeding requiring transfusion of 2 or 3 units of whole blood or packed red blood cells or requiring surgery), and minor bleeding (access site hematoma) (32).

### 2.1 The ProStyle™/ProGlide™ device

The Perclose ProStyle™/ProGlide™ Suture-Mediated Closure and Repair (SMCR) System (Abbott Vascular, Abbott Park, IL, USA) is one of the most recent VCDs able to deliver a single monofilament polypropylene suture and positioning the pre-tied suture knot to the top of the access site. It is indicated for the closure of access sites in the common femoral artery (CFA) using 5 F to 21 F sheaths.

The product's instructions for use (IFU) suggest at least two devices and the pre-close technique for arterial sheath sizes greater than 8 F. No contraindications are known for the use of this device (33). However, some recent clinical studies suggest that a single pre-implanted ProStyle™/ProGlide™ could safely close percutaneous access even in larger sizes access (34, 35).

### 2.2 Access site/anatomical characteristics

Aorto-iliac pathology and femoral accesses anatomical and morphological characteristics were assessed preoperatively by CTA and DUS exams. We considered the mPACSS classification to quantify the vessel calcification (31) (Table 2). In case of plaque with lumen reduction more than 30% and less than 50%, accesses were defined as characterized by moderate atherosclerotic disease.

**Table 2:** mPACSS criteria36.

mPACSS for femoral artery access site's classification	
0 point	None or calcification in <25% of femoral artery TV
+1 point	Calcification in 25%–50% of femoral artery TV
+2 points	Calcification in ≥50% or annular calcification in ≥25% of TV

All vessel measurements were performed using a dedicated software and center lumen line reconstruction from OsiriX MD software—version 12 (PIXMEO, Bernex, Switzerland).

### 2.3 Technique

The standard preimplantation closure technique involved the deployment of pre-implanted ProStyle™/ProGlide™ at the beginning of the procedure. Vascular access was performed under ultrasound guidance, and the CFA was punctured at the centre of its upper wall, in a non-calcified/atheromatous area. The ProStyle™/ProGlide™ device is then mounted on the wire, positioning and deploying at 12 o'clock for a single preimplantation procedure or at 10 and 2 o'clock for a double preimplantation procedure (Control group). Then it is subsequently removed, allowing the ProStyle™/ProGlide™ suture to be deployed. The endovascular procedure is then performed; after the endograft deployment, the sheath is removed, and the ProStyle™/ProGlide™ is tightened over the access wire. The wire is gently tugged to ensure the ProStyle™/ProGlide™ suture has a secure grip around the wire and no bleeding is observed. Two minutes of manual compression is performed for additional safety in all cases.

An ultrasound control is performed at the end of the procedure and a compression bandage is applied for the next 24 h. Patients were re-evaluated with a duplex ultrasound at one-month follow-up.

The procedures were performed by vascular surgeons with extensive experience in the use of ProStyle™/ProGlide™ devices, and operators were the same for both groups.

### 2.4 Statistical analysis

Continuous variables are collected as median and interquartile range (IQR) or mean and standard deviation (SD); categorical data are presented as numbers and percentages. Continuous data were compared with the Mann–Whitney *U* test or *t*-test, whereas categorical data were compared using Fisher's exact or the Chi-square test. A multivariable logistic regression was performed to identify independent predictors (1) sex, (2) sheath diameters, and (3) time of manual compression in comparing bilateral use of a single ProStyle™/ProGlide™ with non-single ProStyle™/ProGlide™ use. Analysis was performed according to major/minor complications criteria, and patients were categorized according to their initial group allocation. All variables with *p*-values < 0.1 is included in the univariate analysis. For all analyses, a two-sided *p*-value < 0.05 was considered significant.

As the entire cohort was highly unbalanced regarding sex distribution, with the aim of obtaining a more homogeneous population for properly comparing, a 1:1 automatic propensity matching was performed using a logistic regression model adjusted for demographic data and comorbidities. The model included all available risk factors listed in the electronic data set.

All analyses were performed using SPSS Statistics version 28.0 [International Business Machines Corporation (IBM Corp.), Armonk, NY, USA].

## 3 Results

### 3.1 Patient population

#### 3.1.1 Prospective study: single-style group (use of only one ProStyle™/ProGlide™ system)

We prospectively enrolled 72 consecutive patients, for a total of 100 vascular accesses, from December 2022 to June 2024, undergoing transfemoral endovascular aorto-iliac procedures with access site closure using a single ProStyle™/ProGlide™ system. EVAR and iliac branch devices used are listed in Table 3.

**Table 3:** Graft used in the two different group.

GRAFT Type	OneStyle Group	Control Group
GORE® EXCLUDER®	11	43
GORE® EXCLUDER® Conformable	7	6
INCRAFT™	4	0
ALTO®	6	5
Endurant™ II/IIs	1	7
TREO®	1	2
MINOS®	1	0
AFX®	0	4
COOK®	0	5
E-TEGRA®	0	1
GORE® EXCLUDER® Iliac Branch	2	6
JOTEC® Iliac Branch	0	2
INCRAFT® Iliac extension	4	0
COOK ZISL® Iliac extension	7	3
GORE® Iliac extension	25	13
ALTO® Iliac extension	8	0
TREO® Iliac extension	7	2
E-TEGRA® Iliac extension	1	1
Endurant™ II/IIs Iliac extension	12	7
AFX® Iliac extension	1	1
MINOS® Iliac extension	2	0

Baseline characteristics, comorbidities, operative reports, complications, and discharge summaries were collected by a thorough review of the individual patient medical charts and grouped by access.

#### 3.1.2 Control group (use of two ProStyle™/ProGlide™ system)

The retrospective analysis collected data from 84 consecutive patients from August 2018 to September 2023 who underwent transfemoral endovascular aorto-iliac procedures with access site closure using two ProStyle™/ProGlide™ systems for a total of 100 accesses.

### 3.2 Data analysis

A total of 156 patients were included in the study. Of those, 72 patients were treated with endovascular aorto-iliac procedure using one single ProStyle™/ProGlide™ system for primary access site closure and 84 patients with two devices (Control group), for a total of 200 endovascular accesses. In 39 patients, a single closure device was used bilaterally.

Single ProStyle™/ProGlide™ and double ProStyle™/ProGlide™ groups were similar for all demographic characteristics except sex, sheath diameters, and chronic kidney disease (CKD) in remote medical history (Tables 4–6). A propensity score matching was used to analyse all these variables and no significant differences were observed between the groups for sex and CKD.

**Table 4:** Baseline characteristics and comorbidities.

Population	OneStyle Group	Control Group	*P* value
Age	73,68 ± 8,46	73,85 ± 6,97	0.877
Male sex (%)	86	96	0.013
Diabetes (%)	6	13	0.091
Hypertension (%)	83	88	0.315
Dyslipidemia (%)	63	54	0.196
CAD (%)	35	32	0.653
CKD (%)	17	7	0.030
Smoke (%)		Data not available	
- Current - Never - Former	55		
	12		
	33		
BMI	25,80 ± 2,87	Data not available	

The underlined values are statistically significant.

**Table 5:** Access site characteristics.

Access site	OneStyle Group	Control Group	*P* value
Calcifications (mPACSS)			0.121
- 0 point - +1 point	85	92	
	15	8	
Thrombosis/Plaque (%)			0.621
- No/Mild - Moderate	92	90	
	8	10	
Previous surgical access	9	4	0.152
Previous endovascular access	8	8	1

**Table 6:** Procedural data.

Procedural data	OneStyle Group	Control Group	*P* value
Technical success (%)	100	100	
Sheat diameter (%)			0.001
12 F	24	17	
13 F	4	0	
14 F	24	4	
15 F	6	4	
16 F	42	75	
Mean sheet diameter (F)	14,38 ± 1,62	15,20 ± 1,52	0.001
Manual Compression (min)	3,81 ± 5,72	2,11 ± 0,85	0.002

The underlined values are statistically significant.

The sheath diameter value was the only factor that was statistically significative at multivariate regression analysis after adjustment for all confounding variables (*p* = 0.001). After adjusting for the initial imbalance in the sheath diameters parameter using propensity score matching, the logistic regression analysis shows that the two groups can be considered clinically comparable, and the sheath diameters value did not have a significant impact on the analyzed outcomes as bleeding and pseudoaneurysm.

No significant differences were found between the two groups regarding the characteristics of the accesses (Tables 5, 6).

Technical success was achieved in all cases with no need for intraoperative open surgical conversion. No further ProStyle™/ProGlide™ or alternative closure systems were required.

Clinical success was achieved in 98% of the accesses when only one ProStyle™/ProGlide™ was used: two cases were followed by minor bleeding (2%), according to the BARC classification, requiring 40-minute manual compression to achieve complete haemostasis. One of the two cases hesitated in a small pseudoaneurysm, which was completely thrombosed at the 48 h DUS control and remained unchanged in size at the 1-month CTA. It completely resolved 6 months after the procedure. No further cases of bleeding, pseudoaneurysms, or arteriovenous fistulas were found on DUS examination in the immediate post-operative and 24 h after the surgical procedure.

Clinical success was achieved in 100% of the cases in the Control Group.

No statistically significant differences were found between the two groups regarding both bleeding (2 vs. 0, *p* = 0,155) and pseudoaneurysm (1 vs. 0, *p* = 0,316) (Tables 7 and 8).

**Table 7:** Access site vascular complications—perioperative.

Access site complications	OneStyle Group	Control Group	*P* value
Bleeding (%)	2	0	0.155
Pseudoaneurysms (%)	1	0	0.316
FAV (%)	0	0	/
Dissection (%)	0	0	/
Open surgical conversion (%)	0	0	/

**Table 8:** Access site vascular complications—24 h.

Access site complications	OneStyle Group	Control Group	*P* value
Bleeding (%)	0	0	/
Pseudoaneurysms (%)	1	0	0.316
AVF (%)	0	0	/
Dissection (%)	1	0	0.316
Open surgical conversion (%)	1	0	0.316

In the prospective group (OneStyle), twenty-two accesses required more than 2 min of manual compression (22%), with an average time of 3, 81 min (Range: 2–40 min) to achieve complete hemostasis. In the two cases of minor bleedings, the suture placed by the closure system probably did not completely close the breach on the artery, so manual compression was necessary to achieve complete haemostasis In the Control group, manual compression exceeding 2 min was needed in only two cases (2/100, 2%), with an average time of manual compression of 2, 11 min (Range: 2–10 min (*p* = 0,002).

**Figure 1:**
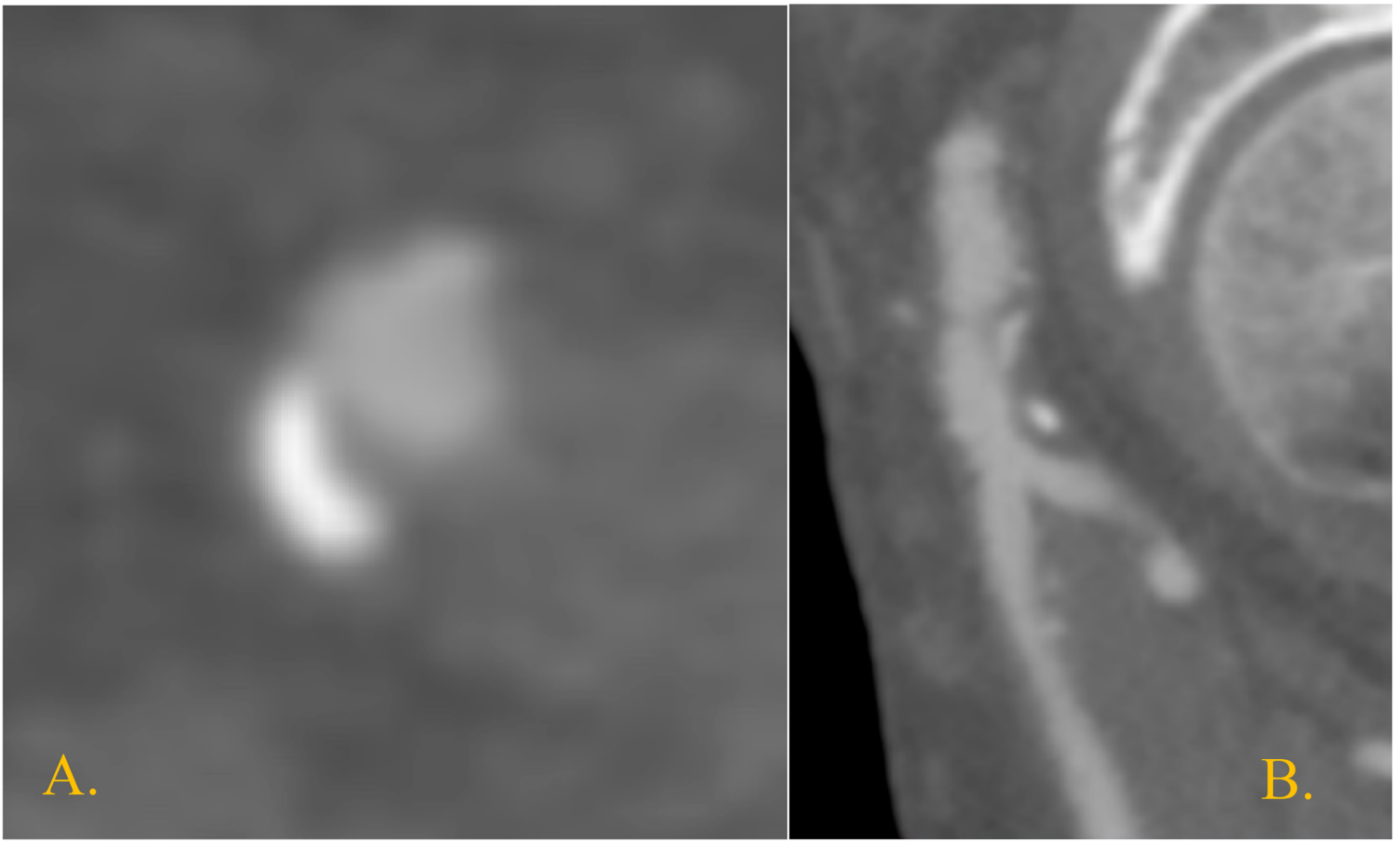
Right CFA at preoperative CTA in the only case of postoperative focal dissection. It shows a posterior atheroma plaque at the level of the endovascular access **(A)** transversal section; **(B)** sagittal section.

**Figure 2:**
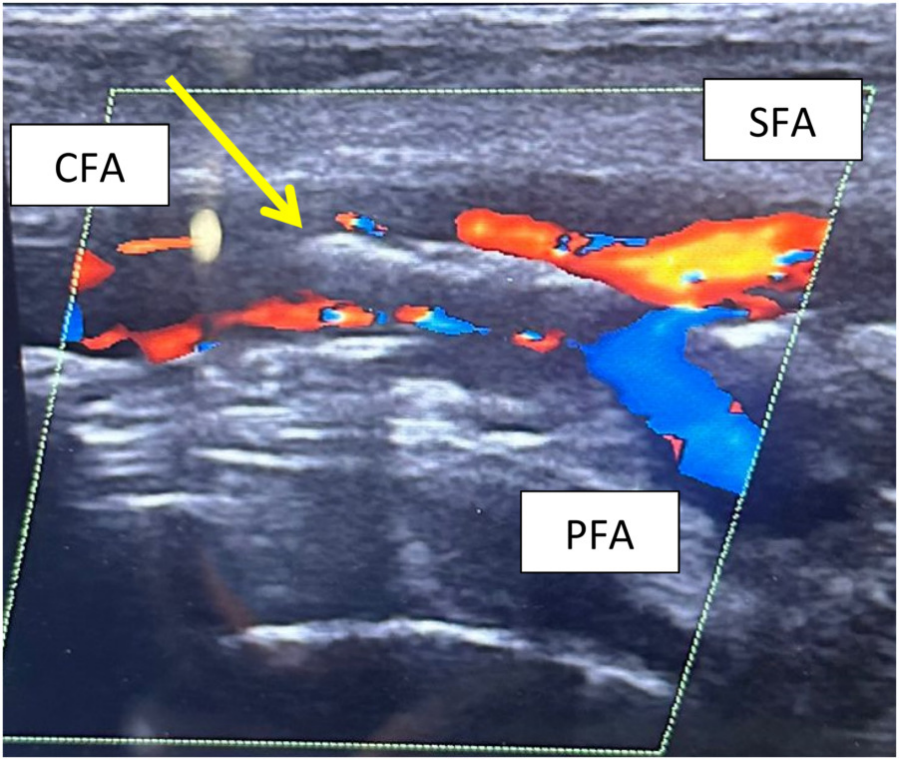
Postoperative CFA dissection at DUS examination. The arrow evidences the double binary sign due to the lifting of the atherosclerotic plaque.

One patient who had closure of the CFA access with only one ProStyle™/ProGlide™ suffered from CFA dissection, which required open endarterectomy (Figures 1, 2).

## 4 Discussion

In our clinical practice, we have successfully utilized the ProStyle™/ProGlide™ system and the pre-close technique for achieving hemostasis at the CFA access during endovascular aortic procedures. Initially, we followed the manufacturer's instructions, deploying one suture knot at a time, and found that a single device was sufficient to ensure complete hemostasis in most cases. This led us to adopt the strategy of using a single ProStyle™/ProGlide™ device for all patients with access sites requiring sheaths smaller than 14 F, since 2019. This approach has proven successful, becoming our standard protocol. Based on this experience, in 2022, we started this prospective study analysing the efficacy of using a single ProStyle™/ProGlide™ in case of sheath up to 16 F. We observed only one case of a pseudoaneurysm, which fully thrombosed within 48 h and reabsorbed spontaneously within six months, and a single case of CFA dissection over a two-year period. However, the dissection was likely caused by the device's suture deployment mechanism, rather than by the use of a single device. In fact, in this patient, the preoperative ultrasound examination revealed moderate atherosclerotic plaque in the femoral artery, which likely led to the suture engaging the plaque instead of the arterial wall, causing a focal dissection. This was managed by femoral endarterectomy due to the patient's claudication symptoms. Such complications, though rare, have been reported in the literature, with a rate of about 1.62% according to Saadi et al. in 2017 (36).

Supporting our results, several studies in the literature have demonstrated the safety and effectiveness of using a single ProStyle™/ProGlide™ device for vascular access closure. In 2020 Krista Dunn et al. (35) reported a retrospective study of 116 patients undergoing EVAR procedure with bilateral percutaneous femoral access. They experienced 6.11% access-site complications: 3.8% required surgical conversions and 2.3% required additional ProGlide™ insertion to achieve adequate hemostasis, although this was not an issue in our study. Even if the median sheath outer diameter was similar to ours (maximum of 18F), they reported a notable number of open surgical conversions, compared to our center results.

Similarly, Taku Ichihashi et al. (34) (2016) studied the use of a single ProGlide™ device in 50 patients undergoing PEVAR, reporting positive outcomes with no surgical corrections needed, though one case required additional manual compression. The sheath diameters in their study were larger than those in the previous study till 22 F sheath, a quite significant data for our discussion.

By the way, larger studies come from interventional cardiology (37), such as those by Kodama et al. (38) (2016), Bazarbashi Najdat et al. (39) (2019) and Joerg Reifart et al. (40), comparing the single-device technique with double-device systems in transcatheter aortic valve implantation (TAVI) procedures. While the single-device approach was generally safe and effective, these studies indicated that some patients required additional closure devices, especially when there were larger sheath sizes or complications related to hemostasis. In contrast, our study showed no such need for additional devices, which suggests a higher success rate in achieving hemostasis with the single-device approach.

In addition, a technique proposed by Gurbhej Singh et al. (41) (2020) involved downsizing the sheath after device deployment to 8 F, before tightening the ProGlide™ suture. This approach increases the procedure time and material use, which did not align with our goal of optimizing efficiency.

Moreover, we documented a single case of isolated CFA dissection, due to the suture deployment mechanism of the ProStyle™/ProGlide™ system rather than the use of a single device. The navigation of the device deployment system, inside the arterial lumen, may cause the plaque to lift and focal dissection when in contact with any atheroma plaques of the common femoral artery. This occurrence is evidenced in Figure 2, where is showed the common femoral focal dissection in post-operative period. This kind of adverse event is reported in literature as a possible complication using this device, as described by Saadi et al. in 2017 whit a rate of 1.62% (36). In our study, only one case was registered out of a total of 200 accesses (0.5%). In our opinion, the role of ultrasound-guided puncture is essential in ensuring safe access to the CFA, minimizing potential complications. The main advantage of our approach is its cost-effectiveness. Using a single device reduces the overall cost compared to the double-device or SEVAR techniques while maintaining the same level of safety and effectiveness. This reduction in device-related costs is significant in the broader context of healthcare economics, potentially improving the affordability of endovascular procedures both locally and globally.

Our monocentric study confirms the possibility to use a single ProStyle™/ProGlide™ closure system for achieving complete hemostasis, with no significant differences in outcomes compared to the double-device approach. Nevertheless, a minor statistical difference in the time for manual compression (only 1. 7 min) was observed, but this did not affect the overall success rate. Our results also suggest a better rate of surgical conversion compared to other studies, with similar positive outcomes for both complex aortic procedures and interventional cardiology treatments.

### 4.1 Limitations

This study is based on a single center experience, with a limited number of patients. Moreover, there is a difference between the two groups in terms of sheeth size although it was not statistically significant in defining outcomes.

However, the sample size reached with the prospective and retrospective cohort of patients enhanced no statistical differences in the outcomes measured. Given the low event rates (e.g., 2% bleeding), the manuscript must acknowledge the limitations in detecting true differences, leading to low statistical power.

However, given the very low complication rate observed, direct comparison with other studies in the literature is challenging.

In order to confirm our findings, the need for further research in randomised or multicentre studies is necessary.

## 5 Conclusions

In conclusion, our study supports the use of a single ProStyle™/ProGlide™ device for hemostasis in endovascular aortic procedures, offering a reliable, cost-effective alternative to more complex strategies, with similar complication-free outcomes and potential for reducing overall healthcare costs.

Total percutaneous endovascular procedures have become an integral part of the clinical practice in an increasing number of centers. These procedures reduce hospitalization time, intensive care unit stay, in hospital readmission and help lower the overall costs of endovascular treatments, which remain.

The results of our study, demonstrate the safety and efficacy of using a Single ProStyle™/ProGlide™ vascular pre-close technique for the closure of vascular accesses up to 16 F, with very low rate of bleedings and pseudoaneurysms, and no cases of open surgical conversion. Employing a single closure system further reduces the cost of endovascular procedures by utilizing fewer materials and expediting the process, without compromising the security of the procedure.

Moreover, the use of the ONESTYLE technique requires no learning curve for professionals already familiar with the ProStyle™/ProGlide™ device, making it a seamless addition to existing practices.

However, our data are limited by the monocentric nature of the study, and the difference in term of sheath sizes between two groups can be considered as a procedural bias in our study. Further research in randomized studies or multicentred studies would be beneficial to fully validate the effectiveness of this technique.

## Data Availability

The raw data supporting the conclusions of this article will be made available by the authors, without undue reservation.
